# MicroRNA-200 Family Members Differentially Regulate Morphological Plasticity and Mode of Melanoma Cell Invasion

**DOI:** 10.1371/journal.pone.0013176

**Published:** 2010-10-04

**Authors:** Ilan Elson-Schwab, Anna Lorentzen, Christopher J. Marshall

**Affiliations:** Cancer Research UK Centre for Cell and Molecular Biology, Institute of Cancer Research, London, United Kingdom; Leiden University, Netherlands

## Abstract

**Background:**

A functional role of microRNAs (miRNAs or miRs) in neoplasia and metastasis is becoming clear, and the miR-200 family has received much attention for potentially regulating tumor progression. The miRNAs of this family have been shown to suppress epithelial-mesenchymal transition, and their down-regulation in some tumors promotes invasion and metastasis. Interestingly, while miR-200 is down-regulated in some cancers, it is up-regulated in others.

**Principal Findings:**

We show that levels of miR-200 are increased in melanoma cell lines compared to normal melanocytes and that miR-200 family members play a role in determining modes of tumor cell migration. Individual tumor cells can invade in either elongated, “mesenchymal-type” or rounded, “amoeboid-like” modes and these two modes of invasion are inter-convertible [1]. In melanoma cell lines, expression of miR-200 members does not suppress invasion but rather leads to a switch between modes of invasion. MicroRNA-200c results in a higher proportion of cells adopting the rounded, amoeboid-like mode of invasion, while miR-200a results in a protrusion-associated elongated mode of invasion. Functional target identification studies suggest that the morphological effects of miR-200c may be mediated by reduced expression of MARCKS, which has been linked to formation of cell protrusions. In contrast miR-200a reduces actomyosin contractility, a feature of rounded morphology.

**Significance:**

Overall our findings call into question the general role of miR-200 in suppressing invasion and metastasis, and highlight novel distinguishing characteristics of individual miR-200 family members.

## Introduction

Melanoma is a highly aggressive human cancer refractory to most treatments. Progression from benign hyperplastic melanocytes to more aggressive disease occurs when tumor cells begin to break down and invade through the basement membrane, and subsequently migrate into the collagen-rich dermis [2]. It has become clear that cancer cells have multiple modes of cell migration during tissue invasion: collective, individual elongated or “mesenchymal-type,” and individual rounded or “amoeboid-type” [1], [3], [4]. The latter two modes of individual cell migration are determined in large part by the balance of Rho and Rac small GTPase signalling. The mesenchymal mode is driven by Rac activation, and involves extensive protrusions and proteolytic activity [1]. The rounded “amoeboid-type” on the other hand is associated with a high degree of actomyosin contractility, membrane blebbing and squeezing through matrices. The amoeboid mode is favored by high Rho/ROCK signalling to elevate actomyosin contractility [1] and is not dependent on extracellular protease activity [4]. Importantly, there is negative feedback between these two signalling pathways, with Rho-kinase inhibiting the mesenchymal mode and Rac inhibiting the rounded form of migration [1]. This interplay allows for dynamic signalling and survival pathway dependence, and plasticity or switching between different morphologies allows cancer cells to invade using distinct pathways to adapt to different environments [4].

Cancer cell morphology can be modulated by microRNA (miRNA or miR) activity [5], [6], [7]. MicroRNAs are 20–24 nucleotide non-coding RNAs that regulate gene expression by targeting the 3′ untranslated region of target mRNA transcripts for degradation and/or translation inhibition. Target specificity is directed by sequence complementarity to the microRNA - particularly in the 2′-8′ seed region - and families have been identified based on miRs that have highly similar or identical seed sequences [8]. Of particular interest in the field of metastasis, the miR-200 family has been shown to regulate epithelial-mesenchymal transition (EMT) and cell migration in a variety of cancer cell lines: miR-200a, -200b, -200c and -141 promote E-cadherin-based junction formation and inhibit cell migration in Boyden-type transwell chambers [9], [10], [11], [12]. It appears that the miR-200 family is targeting Zeb transcriptional regulators [5], particularly Zeb1 [9], [10], [11], [12], [13], preventing the repression of E-cadherin expression by Zeb proteins. Because miR-200 levels are decreased in more aggressive metaplastic breast as compared with ductal tumors [10], and EMT is associated with disruption of cell-cell adhesion and the acquisition of migratory behavior, it has been suggested that the miR-200 down-regulation is involved with the progression of cancer through promoting EMT and cell invasion.

However, while the expression of miR-200 family members is down-regulated in some types of cancer, these microRNAs are over-expressed in other cancers such as melanoma [14], [15], [16], ovarian [17] and colorectal cancers [18]. For example, miR-200c is up-regulated in melanoma lines compared to normal melanocytes [14] and in primary melanoma as compared to benign nevi [16]. Additionally, analysis of microRNA levels showed that miR-200c was up-regulated in melanoma metastases to the lung, although down-regulated in those to the brain, as compared to primary lesions [15]. Taken together, this data suggests that miR-200c is differentially regulated in melanoma and may play a role in disease initiation and/or progression.

In order to investigate the functional effects of the miR-200 family in melanoma, we decided to test whether expression of miR-200 family members affects the ability of melanoma cells to engage in morphological switching and use different modes of migration to invade into a physiologic 3D collagen-I matrix. We confirmed that miR-200 members are up-regulated in melanoma, show they do not suppress invasion into 3D matrices and sometimes increase invasive capacity. Interestingly, while elevation of miR-200a levels led to the mesenchymal mode of cell migration, elevation of miR-200c levels led to the amoeboid mode of migration, highlighting new roles of this microRNA family in switching or plasticity of modes of tumor cell migration.

## Results

### microRNA-200 family members regulate modes of invasion in melanoma cells

**Figure 1:**
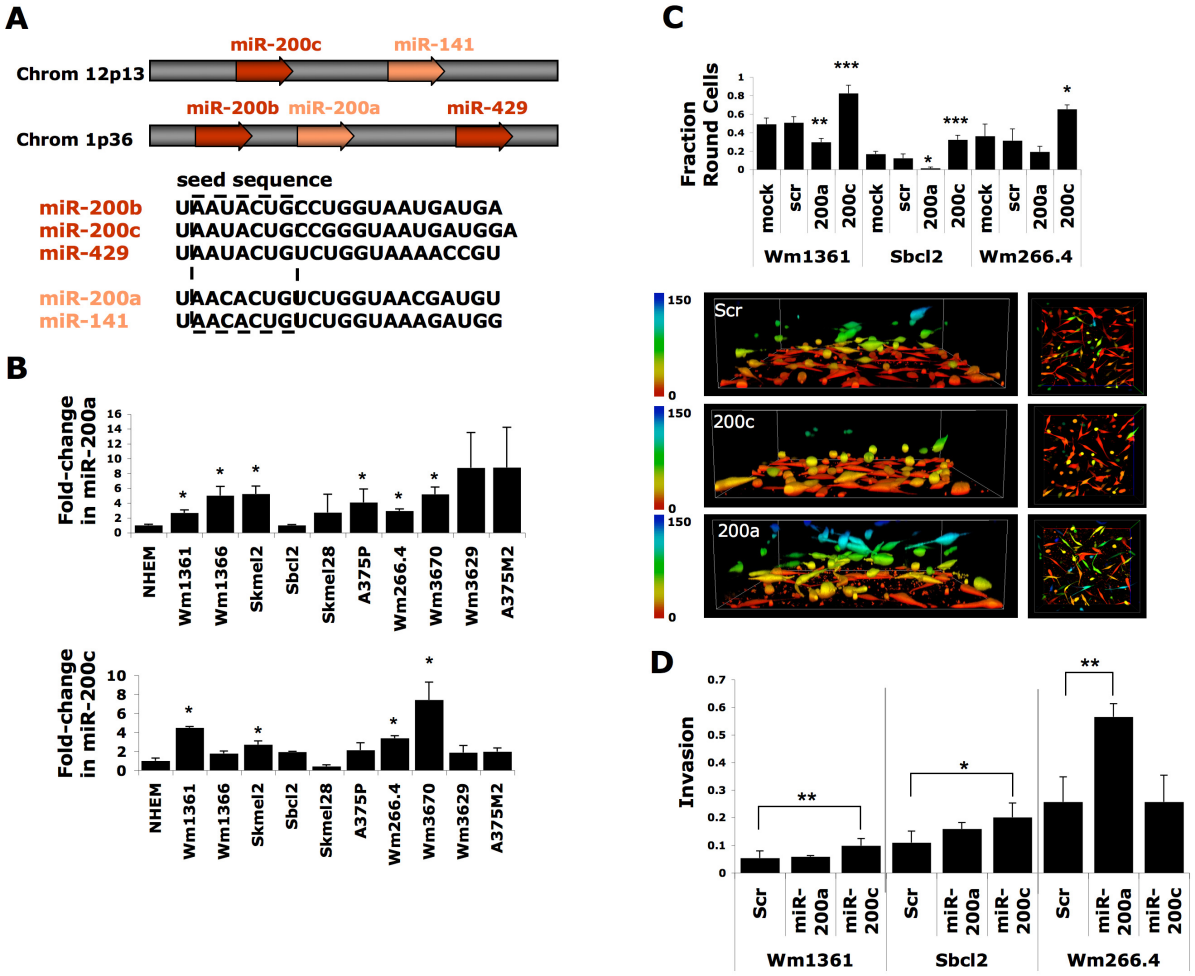
microRNA-200 regulates morphological plasticity of melanoma cells. (A) Schematic representation of the miR-200 family. (B) Relative levels of expression of miR-200a and -200c, normalized to the invariant miR-30b*, in melanoma cell lines compared to normal human epidermal melanocytes. (C) Transfection of miR-200a in Wm266.4 results in an elongated morphology of cells invading into collagen, while miR-200c transfection results in a rounded morphology (scale bars in um). Quantification of the percentage of cells with rounded morphology for melanoma cell lines invading into collagen. (D) miR-200 family members do not inhibit melanoma cell invasion, but can promote invasion. Each experiment was performed at least three times and representative examples are shown. Error bars represent ± SEM; unpaired t-test *p<0.05 **p<0.01 ***p<0.001.

Individual members of the miR-200 family can be subdivided in two different ways according to genomic location or primary sequence (Fig. 1A). We selected for our studies miR-200a and -200c because each represents distinct subclasses according to seed sequence and genomic location. In order to examine expression of miR-200 members in melanoma, we examined expression of miR-200a and -200c using quantitative PCR in a panel of melanoma cell lines compared to normal human melanocytes using miR-30b* as a control as we found its levels were relatively invariant across the melanocytes and melanoma cell line panel (Fig. S1). For these studies the cells were plated on a thick deformable layer of collagen-I because this substrate mimics aspects of a true 3D matrix and represents a more physiologic environment for studies of melanoma cell migration [1]. We have previously shown cell behaviour on this matrix closely correlates with results obtained with invasion assays into a 3-dimensional matrix [1]. As shown in Figure 1B, many of the melanoma lines examined had significantly higher levels of both microRNAs than primary normal human melanocytes plated on the same matrix.

Many studies have shown that miR-200 family members inhibit cancer cell migration [9], [10], [11], [12]. Melanoma cell invasion in vivo is influenced by cell morphology and switching between different modes of invasion [1], [19]. The rounded “amoeboid” form of movement seen in vivo is not revealed by assays of cell migration on rigid 2D substrates, or transwell assays where cells have to migrate through pores coated with a thin layer of extra-cellular matrix [3], [4]. Hence in order to examine cellular morphology of invading cells, various melanoma lines (Wm1361, Wm266.4 and Sbcl2) were either mock transfected or transfected with scrambled control, miR-200a or miR-200c, allowed to invade into a thick layer of collagen, and imaged using confocal microscopy. As we have observed previously [1], different melanoma cell lines exhibit varying mixtures of invaded cells with an elongated or round morphology. Expression of miR-200c, however, significantly increased the percentage of cells utilizing the rounded mode of invasion (Fig. 1C). This effect on cell morphology is most pronounced by viewing the plane perpendicular to the axis of invasion with depth indicator coloring (Fig. 1C, right), which shows that the invaded miR-200c-transfected cells (yellow and green) are significantly rounder than control cells. Quantification of the proportions of cells exhibiting different modes of invasion inside the collagen layer reveals that miR-200c induces up to a 2-fold increase in the proportion of invaded round cells in multiple melanoma cell lines (Fig. 1C, because mock and scrambled results were highly similar across experiments, only scrambled control is presented for other experimental data.) Rounded morphology was induced by transfection of miR-200c in multiple melanoma cell lines when plated on top of a thick deformable layer of collagen (Fig. S2). Interestingly, miR-200c transfection of primary human melanocytes, which have low levels of miR-200c (Fig. 1B), results in adoption of a round morphology (Fig. S2).

In contrast to the results obtained with miR-200c, melanoma cells transfected with miR-200a invaded collagen-I in an elongated manner often associated with the formation of multiple protrusions (Fig. 1C). This elongation-promoting effect of miR-200a was observed in multiple melanoma lines invading into 3D collagen-I matrices (Fig. 1C), and also in the case where cells are plated on top of a thick collagen matrix (Fig. S3). Many papers report that miR-200 members from different subfamilies have inhibitory activities on cell migration or tumor-progression [9], [10], [11], [12]. The data presented here suggest that different miR-200 family members do not inhibit migration but have distinct effects on the mode of tumour cell migration when studied by invasion into a 3D collagen-I matrix, a more physiological model of the interstitial matrix. Therefore different family members should be considered independently of each other, as also noted by others [13], [20].

Previous observations that miR-200c expression suppresses cell migration [9], [10], [11], [12], are difficult to reconcile with the apparent up-regulation of miR-200 members in migratory melanoma and melanoma cell lines. In order to examine whether miR-200 family members affect the capacity of melanoma cells to invade, we quantified invasion in the 3D collagen invasion assay [21], calculating invasion index measured as a ratio of the number of cells invaded at 50 um divided by total cells. As shown in Figure 1D, transfection of melanoma lines with synthetic miR-200c resulted in either no change in invasion index in the Wm266.4 cell line, or a 2-fold activation of invasive capacity in Wm1361 and Sbcl2. The invasion of Wm266.4 cells was strongly promoted by miR-200a, however, and was increased 15-fold compared to controls in conditions of reduced serum (data not shown). No significant change in invasion was observed with miR-200a in Wm1361 or Sbcl2. Thus while there is a clear-cut effect of miR-200a and miR-200c on the mode of invasion in all cell lines tested, only in some cell lines is there also an overall stimulation of the number of invaded cells. The mechanisms underlying these differences are unclear, but these observations show that in some situations, miR-200 family members not only alter the mode of invasion, but can also stimulate invasion.

Levels of miR-200 family members were compared across cell lines in order to ascertain whether there is a simple relationship between levels of miR-200, morphology, and mode of invasion. Of note, Sbcl2, which invades in a highly elongated manner, has lower levels of miR-200c as compared to Wm1361 and Wm266.4, both of which display a higher percentage of cells with rounded morphology. Levels of miR-200a show a similar pattern to miR-200c, suggesting that miR-200c may be the more important driver of melanoma cell morphology. However there is no clear correlation between miR-200 levels and morphology across a larger panel of melanoma lines, implying that the complexity of the network of players responsible for driving melanoma cell plasticity is not fully appreciated. Neither is there a correlation between levels of expression of miR-200 family members and invasive capacity, other than in normal human melanocytes, which have very low miR-200 levels and are not invasive. Thus there is no simple relationship between miR-200 levels and invasion apparent from these experiments.

### miR-200c regulates MARCKS

**Figure 2:**
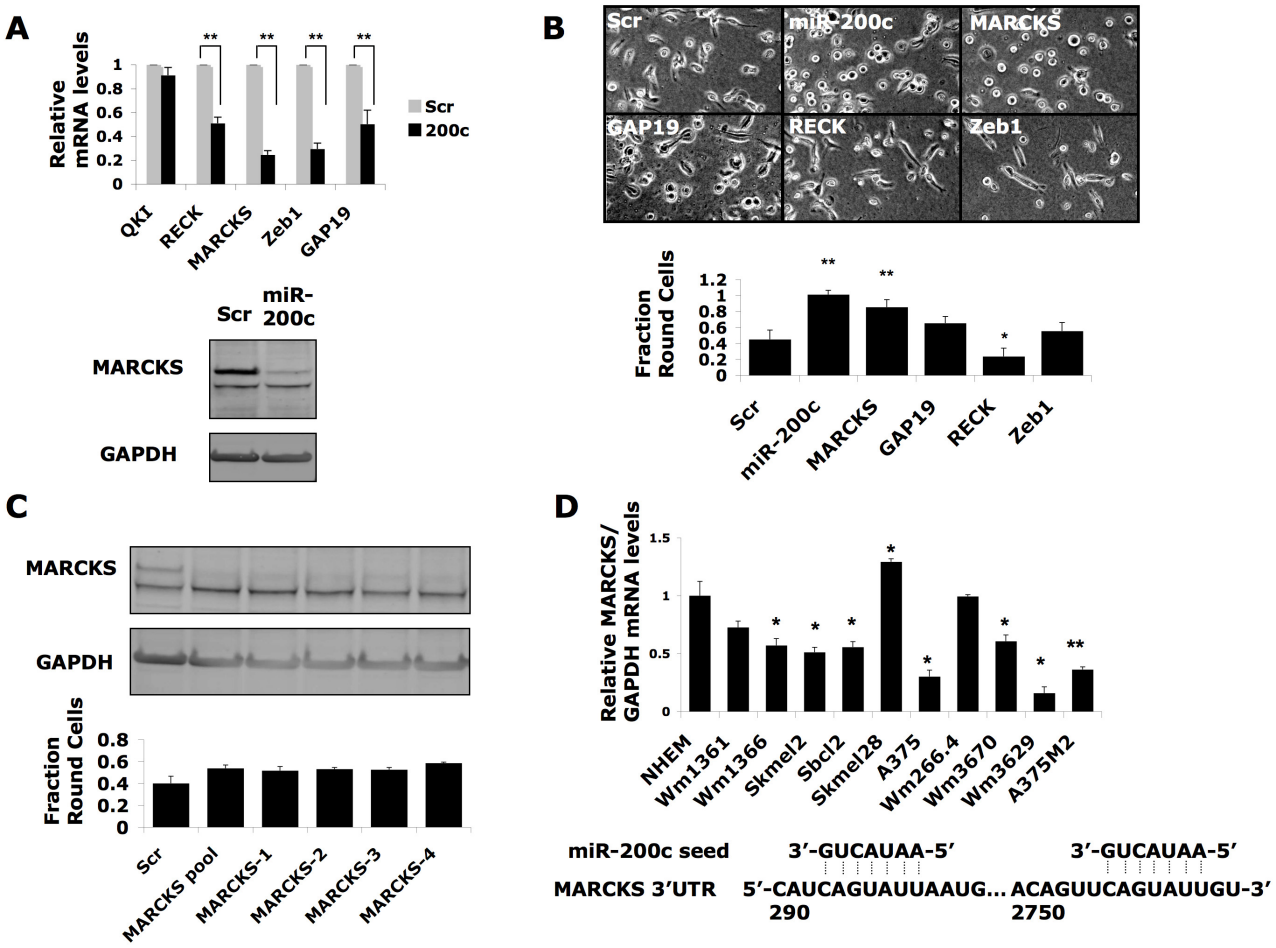
microRNA-200c regulates MARCKS. (A) The effect of miR-200c transfection on mRNA levels of predicted target genes in Wm266.4 melanoma cells was assessed using GAPDH as a control. Lower panel: immunoblot of MARCKS expression. (B) Brightfield imaging and morphological quantification of Wm266.4 cells transfected with siRNAs targeting miR-200c-responsive genes. (C) MARCKS knock-down by four individual siRNAs leads to rounded cell shape in Wm266.4 cells. (D) Levels of MARCKS mRNA in melanoma cell lines and normal melanocytes, measured by qPCR and normalized to levels of GAPDH. Error bars represent ± SEM; unpaired t-test *p<0.05 **p<0.01.

As up-regulation of miR-200c levels in melanoma cells leads to a shift to the rounded mode of invasion, we sought to learn how this microRNA induces round cell morphology. A candidate target gene list was first compiled by cross-referencing published miR-200c-responsive genes [12], [22], [23] with genes previously identified to play a role in cell shape and cancer, together with targets identified by the in-silico prediction programs TargetScan [8] and TargetRank [24]. The Wm266.4 line was selected for studies on potential target genes because previous work has shown that these cells readily interconvert between rounded and elongated morphologies and invade as mixtures of similar proportions of rounded and elongated cells [1], [3]. This plasticity makes Wm266.4 a system useful for identifying factors regulating cell morphology and mode of invasion. Because mRNA levels are a good indicator of microRNA activity [25], [26], quantitative PCR was performed for the candidates genes in Wm266.4 cells transfected with miR-200c or scrambled microRNA negative control. As shown in Figure 2A, mRNA levels of four predicted target genes (using GAPDH levels as a control) were decreased by miR-200c: reversion-inducing-cysteine-rich protein with kazal motifs (RECK), Rho GTPase activating protein 19 (ARHGAP19), myristoylated alanine-rich protein kinase C substrate (MARCKS), and zinc finger E-box binding homeobox 1 (Zeb1/TCF8), while levels of the predicted target quaking homologue (QKI) were not affected.

In order to test whether reducing expression of any of these miR-200c responsive genes results in rounded cell morphology, the genes were knocked down (Fig. S4) by RNA interference using oligonucleotide pools and cells were plated on top of a thick layer of collagen for imaging. While control Wm266.4 cells consist of an approximately equal mixture of elongated and round cells under these conditions, a 2-fold increase in the percentage of rounded cells was observed in cells transfected with MARCKS siRNA, like those transfected with miR-200c (Fig. 2B). Importantly there was no significant change in morphology of cells transfected with Zeb1 siRNA that has previously been shown to result in reduced cell migration in 2D migration assays [9]. Knockdown of GAP19 showed no statistically significant effect on cell morphology while knockdown of RECK reduced cell rounding suggesting that in the morphological switch driven by miR-200c, the reduction of MARCKS levels by miR-200c is dominant over the effects of RECK reduction.

Because it produced a robust effect on cell morphology, MARCKS knockdown was further studied by immunoblotting and deconvolution of the siRNA pool used in Figure 2B. Immunoblotting confirmed that miR-200c efficiently knocks down MARCKS at the protein level (Fig. 2A). Furthermore, four individual siRNAs that decreased MARCKS protein levels also induced the rounded morphology (Fig. 2C). These results are in accordance with the demonstrated role of MARCKS in the formation of protrusions and dynamic adhesions [27] and are observed in Sbcl2 and Wm1361 melanoma lines as well as Wm266.4 (Fig. S4). Thus it appears that MARCKS is a key target of miR-200c and its reduced expression contributes to a rounded morphology.

MARCKS has multiple predicted miR-200c binding sites (Fig. 2D). In order to identify a possible relationship between MARCKS and miR-200c levels in melanoma, MARCKS levels were investigated in the melanoma cell line panel and normal melanocytes plated on a physiologic collagen matrix. As shown in Figure 2D, MARCKS is down-regulated in most melanoma lines compared to control melanocytes, demonstrating the opposite relationship to that seen for miR-200c (Fig. 1B). Notably Skmel28, which has the highest level of MARCKS, has a very elongated morphology on collagen, and is the melanoma line with the lowest level of microRNA-200c. However, the reciprocal relationship of increased miR-200c and decreased MARCKS levels compared to control melanocytes only reaches statistical significance for Skmel2, and Wm3670. This suggests that there are additional players to miR-200c that are contributing to MARCKS regulation.

**Figure 3:**
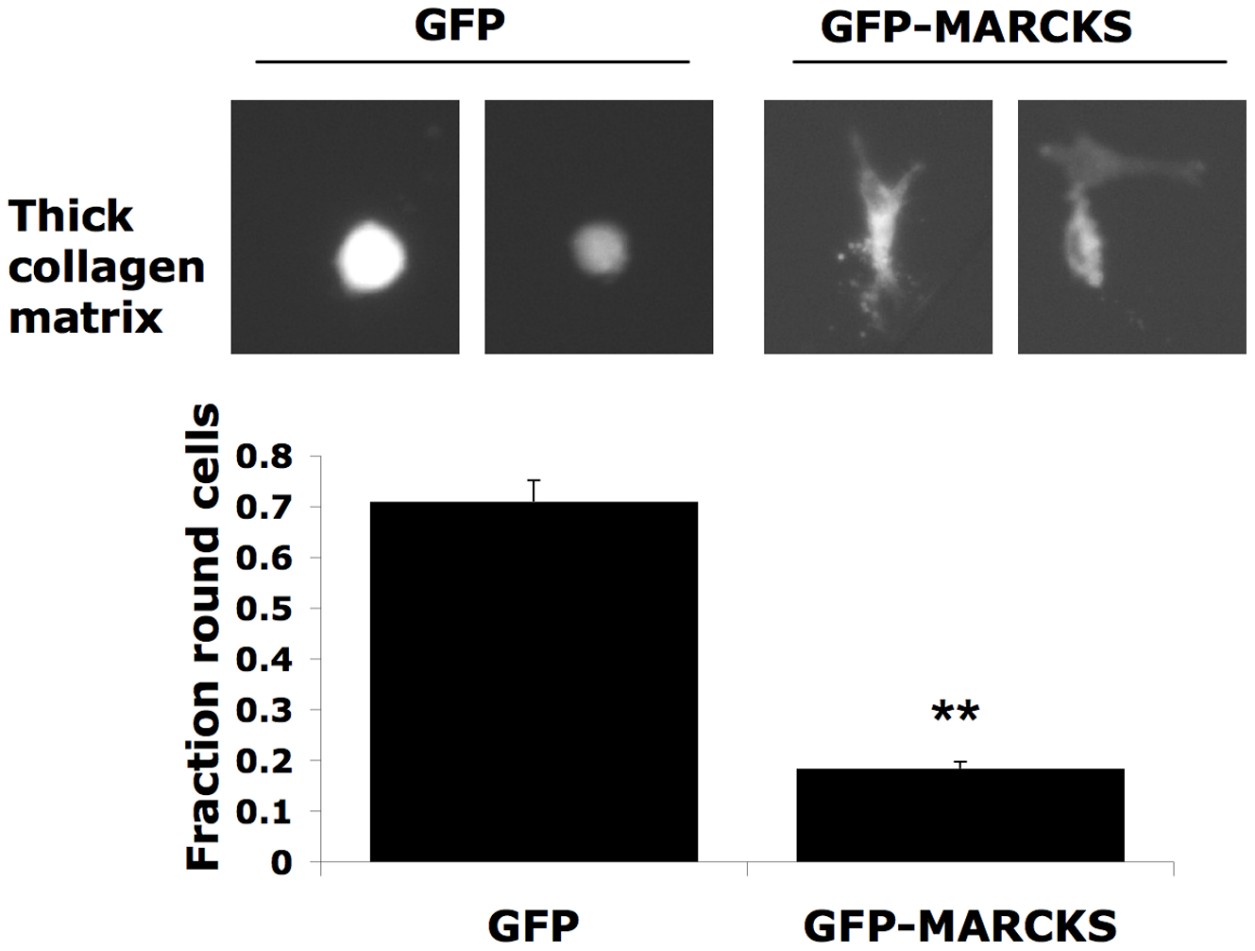
MARCKS expression leads to protrusion formation in melanoma cells. Images of GFP-MARCKS or GFP expressing Wm266.4 cells plated on a thick layer of collagen-I. Error bars represent ± SEM; unpaired t-test **p<0.01.

In order to examine the effects of MARCKS over-expression, a previously published MARCKS-gfp construct [27] was transfected into Wm266.4 cells. Cells that express the fusion construct plated on a thick layer of pliable collagen matrix had a more elongated shape than cells transfected with a GFP control plasmid (Fig. 3), further supporting the role of MARCKS in modulating melanoma cellular morphology.

### miR-200a targets

**Figure 4:**
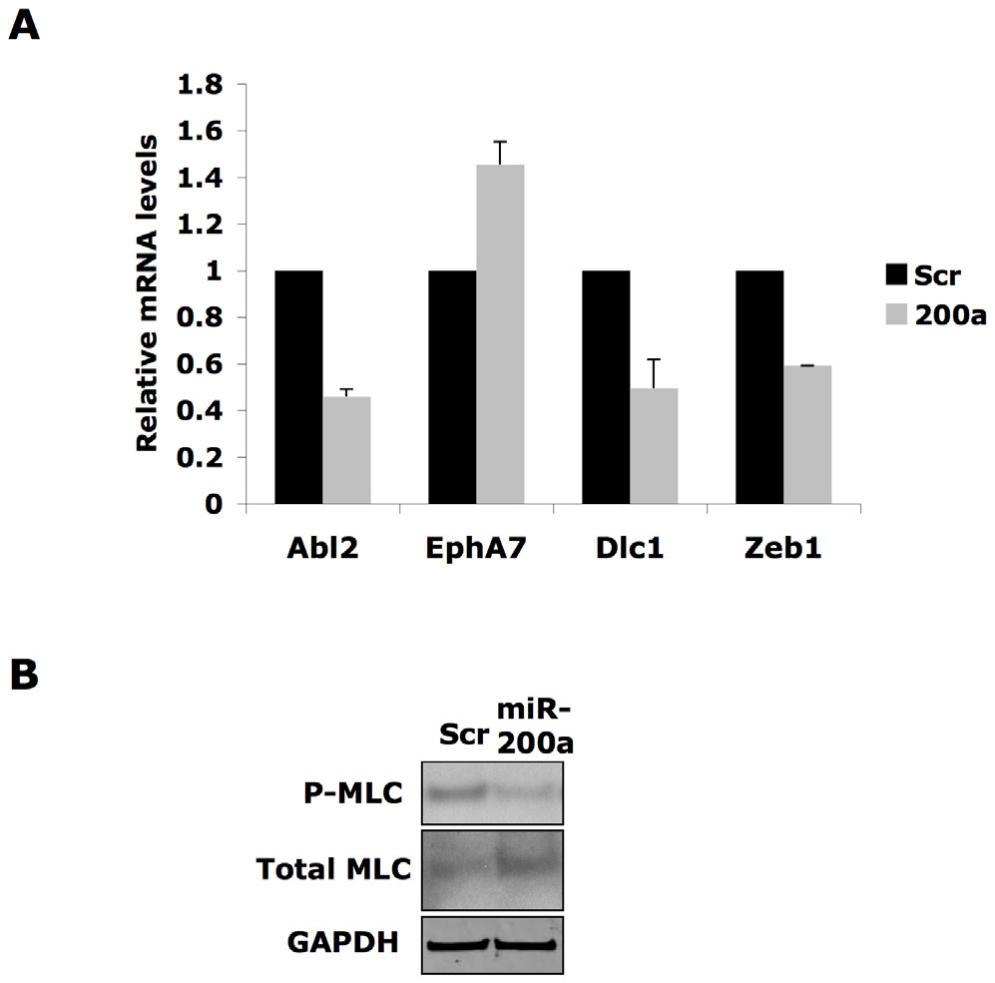
miR-200a associated elongation is associated with decreased actomyosin contractility. (A) The effect of miR-200a transfection on mRNA levels was measured for predicted target genes in Wm266.4 melanoma cells using GAPDH as a control. (B) Transfection of Wm266.4 cells with miR-200a leads to decreased pMLC levels.

Unlike miR-200c, transfection of miR-200a led to a switch to elongated morphology and mode of invasion. Initial tests were performed to assess the effect of miR-200a transfection on mRNA levels of potential targets. Genes with putative miR-200a targeting sequences that have been implicated in cell migration and cancer include Abelson murine leukemia viral oncogene homolog 2 (Abl2), deleted in liver cancer 1 (Dlc1), Eph receptor A7 (EphA7), and Zeb1. Transfection of Wm266.4 cells with either scrambled control or miR-200a shows that miR-200a down-regulates Abl2, Dlc1, and Zeb1, but not EphA7, as measured by quantitative PCR (Fig. 4A). Zeb1 knockdown results in no statistically significant change in cell morphology and attempts at siRNA targeting of Abl2 and Dlc1 produced inconclusive data on cellular morphology due to confounding off-target effects (data not shown).

Actomyosin contractility is an important determinant of tumor cell morphology and is regulated by myosin light chain (MLC2) phosphorylation [1]. Tumour cells with high levels of actomyosin contractility have a round morphology in 3D collagen matrices and inhibition of signaling to MLC phosphorylation or direct inhibition of myosin II by blebbistatin leads to an elongated morphology [1], [28]. In order to determine whether miR-200a regulates cell shape through modulation of actomyosin contractility, levels of MLC2 phosphorylation were examined in lysates of Wm266.4 cells transfected with miR-200a or scrambled oligonucleotides and plated on a thick layer of collagen. As shown in Figure 4B, miR-200a leads to a marked decrease in levels of phosphorylated MLC2, suggesting that this microRNA may be promoting an elongated phenotype via reducing actomyosin contractility.

## Discussion

The results presented here suggest that the miR-200 family of microRNAs regulates melanoma cell morphology and associated invasion, and that functional differences exist between different miR-200 family members. Deregulation of miR-200 expression has been observed in multiple cancer studies, and this group of microRNAs has been shown to be up-regulated in some, but down-regulated in other types of cancer. The majority of functional studies, particularly in breast cancer have concentrated on roles of down-regulation, suggesting that miR-200 members prevent tumor progression by negatively regulating Zeb transcriptional repressors [9], [10], [11], [12] and consequently maintaining E-cadherin junctions and preventing EMT. Up-regulation of miR-200 members has however been identified in cancer of the ovaries [17], cervix [29], bowel [18], melanocyte lineage [14], [15], [16], bile duct [30], and prostate [31] as well as in cancer models [32], [33], [34], [35]. These findings suggest that miR-200 regulation and activity may be highly context-dependent and that miR-200 may be promoting progression of some cancers. Two recent papers lend weight to this argument in showing that cell proliferation in culture [36] and metastasis in vivo [37] are promoted by miR-200 expression. The data presented here supports a role for the miR-200 family in melanoma cell invasion as both miR-200a and miR-200c are up-regulated in melanoma lines and drive different modes of invasion into a physiologic collagen-I matrix.

Two miR-200 seed classes have been identified for the two subfamilies, miR-200bc/429 and miR-200a/141, based on one non-identical base within the seed region (Fig. 1A), and distinctive target predictions for these subfamilies are produced by algorithms such as TargetScan which take into account conservation of microRNA-target UTR complementarity across species. However, many reports have shown the different members of the miR-200 family have similar effects on epithelial-mesenchymal transition (e.g. through the coordinated repression of Zeb proteins) [9], [10], [11], [12]. Recently functional differences between these subclasses of miR-200 have been identified [13], [20], and our findings demonstrate that miRs from different subfamilies have dramatically different effects on cellular morphology in 3D collagen invasion assays. Thus variation in the expression of miR-200 family members in melanoma could lead to plasticity of cell morphology and of mode of migration, conferring on tumor cells the capacity to cope with different milieus in the tumor microenvironment. Previous work shows that the rounded mode of cell movement is favoured at the edge of tumors while within the tumor, tumor cells move in an elongated fashion [1]. The ability of miR-200 to regulate morphological plasticity may be important after dissemination from the tumor as it has been shown that miR-200c levels are different in melanoma samples located at primary versus distant metastatic sites [15].

We show that expression of miR-200c in melanoma cells drives the rounded-amoeboid form of cell migration and MARCKS down-regulation. MARCKS is a peripheral membrane protein whose translocation to the cytosol is mediated by PKC-dependent phosphorylation [38]. The PKC-MARCKS axis has been shown to promote dendritic spine formation in hippocampal neurons [39] and formation of protrusive adhesions in the highly motile Wm1617 melanoma line [27]. We show MARCKS knockdown - using transfection of siRNA or of miR-200c - inhibits protrusion formation and results in rounded “amoeboid” morphology. These results with knockdown of MARCKS parallel our previous observations with inhibiting signalling by Rac or its effector WAVE2; inhibition of Rac-driven protrusions results in a round morphology and conversion to the rounded “amoeboid” mode of migration [1]. Thus there seems to be a very tight coupling between inhibiting protrusion formation and adoption of the rounded “amoeboid” mode of migration. Interestingly MARCKS is inactivated in small intestinal adenocarcinoma [40] and decreased MARCKS levels are observed in transformed cell lines [41]. Moreover, over-expression of MARCKS in cancer cells results in decreased proliferation [42] while knockdown has been shown to increase migration [43].

We also show that invasion is not inhibited by miR-200 family members in a physiologic 3D matrix environment, which is in contrast to observations of others using rigid transwell migration chambers [9], [10], [11], [12]. In contrast to transwell studies where cells have to migrate through a pore in a rigid membrane thinly coated with a matrix protein, we have used thick deformable collagen-I matrices where cells can adopt either the rounded “amoeboid-type” of movement or elongated mesenchymal-type mode of movement [1], [4]. The conditions of the 3D assay are more akin to migration through the interstitial environment because the cells are surrounded by matrix and the deformability of the matrix means that cells can use their contractile force to deform and squeeze through the matrix [1], [4]. Interestingly in the 3D assay miR-200a and miR-200c can enhance invasion in some cell lines (Fig. 1D). Since both miR-200a and miR-200c can enhance invasion but have opposite effects on morphology it is clear that there is no simple relationship between mode of invasion and total invasion.

Preliminary studies to find targets of miR-200a have identified multiple genes that are targeted by this microRNA (Fig. 4A), and represent potential mediators of the miR-200a-induced effects observed in culture. Previous reports have shown that MLC2 phosphorylation is associated with the rounded cell morphology [1], [28], and our finding that phosphorylation of MLC2 is altered by miR-200a suggests actomyosin contractility is regulated by miR-200a (Fig. 4B). As previously shown [1], [44], actomyosin contractility and rounded cell morphology are positively regulated by RhoA-dependent ROCK signalling. This axis also inhibits Rac-associated protrusive migration, and thus the Rho/Rac system may represent a direct or indirect target of miR-200a.

It would be desirable to knockdown these microRNAs to further demonstrate their role in invasion and cellular morphology, however thus far, multiple attempts at miR-200 knockdown - using synthetic and expression-based methods - have proven unsuccessful. Quantitative PCR analysis revealed that treatment with inhibitors towards miR-200c, for example, fails to efficiently knockdown the microRNA, as assessed by mRNA levels of the miR-200c-targets Zeb1 and MARCKS (data not shown). It has been noted that miR-200b-induced rounding in colon cancer cells was refractory to microRNA antagonists [6], and perhaps it is difficult to achieve microRNA knockdown in some cell lines. The data presented here nevertheless support a role of miR-200, which is over-expressed in melanoma, in promoting melanoma cell plasticity and associated invasion.

It is interesting that the miR-200 family seems to be up-regulated and promoting tumor progression in some circumstances, yet down-regulated in others. Tissue of origin may account for this observation, however another possibility is that miR-200 performs this dual-act within the same type of cancer. Previous studies have focused primarily on the epithelial-mesenchymal transition, a point of restriction towards individual cells breaking off from epithelial and E-cadherin-based tumor structures, while our studies model different modes of single-cell invasion. It is possible that miR-200 levels are differentially regulated at different times in tumor progression, facilitating distinct outcomes.

In conclusion, we find the miR-200 family can play multiple roles in cancer including promotion of invasion and switching between modes of invasion. Additionally, our data show that miRs from different miR-200 subfamilies have opposing effects on morphological modes of invasion, highlighting novel distinguishing activities of individual miR-200 family members in melanoma.

## Methods

### Cell Culture

Normal human epidermal melanocytes (Invitrogen C-102-5C) were grown in 254 Media (Invitrogen M-254-500) and supplement (Invitrogen S-002-5). A375P and A375M2 cells were obtained from Dr R. Hynes (Howard Hughes Medical Institute, Massachusetts Institute of Technology). SkMel2, SkMel28, Sbcl2, Wm1361, Wm1366, Wm266.4, Wm3629, Wm3670, and 501Mel were obtained from Drs. I Arozarena and R Marais (Institute of Cancer Research, London). Melanoma lines were maintained in Dulbecco's modified Eagle's medium (DMEM) containing 10% fetal calf serum. A 50∶50 mixture of supplemented 254 media and DMEM-10% was used for the miR-200c transfection of melanocytes.

### Transfection

Individual and pooled siGENOME siRNAs (Dharmacon) and Pre-miR™ miRNA Precursor Molecules and scrambled negative control #1 (Ambion) were diluted to 10nM in Opti-Mem (Invitrogen) and stabilized with INTERFERin (Polyplus) prior to transfection onto cells containing DMEM with 10% serum. Plasmid transfection of GFP-MARCKS – a generous donation from Dr. Karl Pfenninger [27] – and miR-200 transfection of normal melanocytes were performed using Lipofectamine2000 and Lipofectamine (Invitrogen), respectively, following the manufacturer's protocol.

### Cell Culture on Collagen

Fibrillar bovine dermal Purecol collagen (Nutacon) was prepared at a 1.7 mg/ml dilution and 300 ul was placed in wells of 24-well plates and allowed to polymerize at 37C for 3 hours or overnight. The thin coating was obtained by aspirating all but a thin film of collagen from the well before polymerization. Cells were seeded on top of collagen in medium containing 10% serum and allowed to adhere for 24 hr, at which point media was switched for DMEM with 1% serum. Except where indicated, all cell imaging, immunoblotting, and RNA isolation was performed on cells cultured on a thick layer of collagen.

### RNA Isolation, Measurement and Quantitative PCR

RNA was harvested using Trizol (Invitrogen) according to the manufacturer's instructions. Quantification of total RNA was performed using a Nanodrop spectrophotometer (Thermo Scientific, UK). Analysis of specific microRNA levels was performed following the manufacturer's instructions using TaqMan MicroRNA Reverse Transcription Kit, and TaqMan 2x Universal PCR Master Mix, No AmpErase UNGb (Applied Biosystems PN 4366596 & PN 4324018). Specific microRNA primers used were for miR-200a, miR-200c, and miR-30b* (Applied Biosystems 000502 4427975, 002300 4395411, and 002129 4395240). The Quantitect SYBR Green RT-PCR system was used for analysis of mRNA transcript levels, according to the manufacturer's instructions (Qiagen). GAPDH was measured as the mRNA loading control (Qiagen QT01192646).

### Antibodies & Immunoblotting

Antibodies used in the study included: MARCKS (Santa Cruz sc-6455), p-Thr18/Ser19-MLC2 (Cell Signalling 3674S), MLC total (Sigma), GAPDH (Novus NB300-221). Lysates were fractionated by SDS-PAGE and transferred to nitrocellulose membranes. Western blotting was performed with the ECL Plus detection System (GE Healthcare) with horseradish peroxidase-conjugated secondary antibodies (Sigma) or using a fluorescent secondaries and imaging system (Li-Cor). For p-Thr18/Ser19-MLC2 detection, whole-cell extracts from cells on collagen were harvested in Laemmli sample buffer and sonicated for 15 s prior to centrifugation.

### Invasion

The 3-dimensional collagen invasion assay was performed as described [1], [21]. DMEM media with 5% serum was used for stimulus. Invasion is measured as a ratio of cells invaded to 50 um over total cells.

### 3-Dimensional Confocal Imaging

For 3-dimensional imaging of invaded cells, the invasion assay was performed as above except it was set up in ibiTreat u-Plate 96-well plates (Ibidi 89626). Z-stacks of GFP-expressing invading cells were obtained by indirect immunofluorescence microscopy using a Zeiss LSM710 confocal microscope. Three-dimensional reconstructions were made with Zen software (Zeiss). For evaluation of the percentage of elongated cells invading, a cell was considered elongated when its longest dimension was twice the shortest and when it showed at least one protrusion.

### microRNA Target Prediction

TargetScan [8] and TargetRank [24] were used to predict microRNA targets.

### Statistical Analysis

All experiments were repeated at least 3 times and representative data/images are shown. Student's t test was performed to determine statistical significance. Error bars indicate +SEM.

## Supporting Information

Figure S1 miR-30b* serves as a control for microRNA expression. Expression levels of miR-30b*, standardized to total RNA measured by Nanodrop spectrophotometry, in melanoma cell lines and normal human melanocytes.
(0.39 MB TIF)

Figure S2 Rounded cell morphology is induced by miR-200c in melanoma cell lines and normal human melanocytes plated on a thick collagen matrix. Cells was transfected with either a negative control or miR-200c, plated on a thick collagen matrix, and examined for morphology. (A) Left Panel: Brightfield images of Wm266.4 cells. Right Panel: Quantification of the fraction of round cells following transfection with miR-200c in 7 melanoma cell lines. Error bars represent ± SEM; unpaired t-test *p<0.05, **p<0.005, ***p<0.0001. (B) Brightfield images of normal human melanocytes.
(1.86 MB TIF)

Figure S3 miR-200a induces elongation in melanoma cell lines on a thick collagen matrix. Cells were transfected with either a negative control or miR-200a, plated on a thick collagen matrix, and examined for morphology. Upper panel: Brightfield images of Wm266.4 cells. Lower: Quantification of the fraction of round cells following transfection with miR-200a in 7 melanoma cell lines. Error bars represent ± SEM; unpaired t-test *p<0.05, ***p<0.0005.
(1.78 MB TIF)

Figure S4 siRNA knockdown of miR-200c targets. (A) Quantitative PCR analysis, using GAPDH as a control, confirms gene knockdown by siRNAs in Wm266.4 cells imaged in Figure 2. (B) Upper: Quantification of the fraction of rounded cells following knockdown of MARCKS by siRNA or miR-200c, in Wm1361 and Sbcl2 melanoma cell lines. Lower panel: immunoblot of MARCKS expression following transfection of miR-200c or siRNA targeting MARCKS.
(0.65 MB TIF)
