# Supplementary figures and images for: MicroRNA-200 Family Members Differentially Regulate Morphological Plasticity and Mode of Melanoma Cell Invasion
Source: PLoS One. 2010 Oct 4;5(10):e13176. doi: 10.1371/journal.pone.0013176 (PMC2949394; doi:10.1371/journal.pone.0013176)

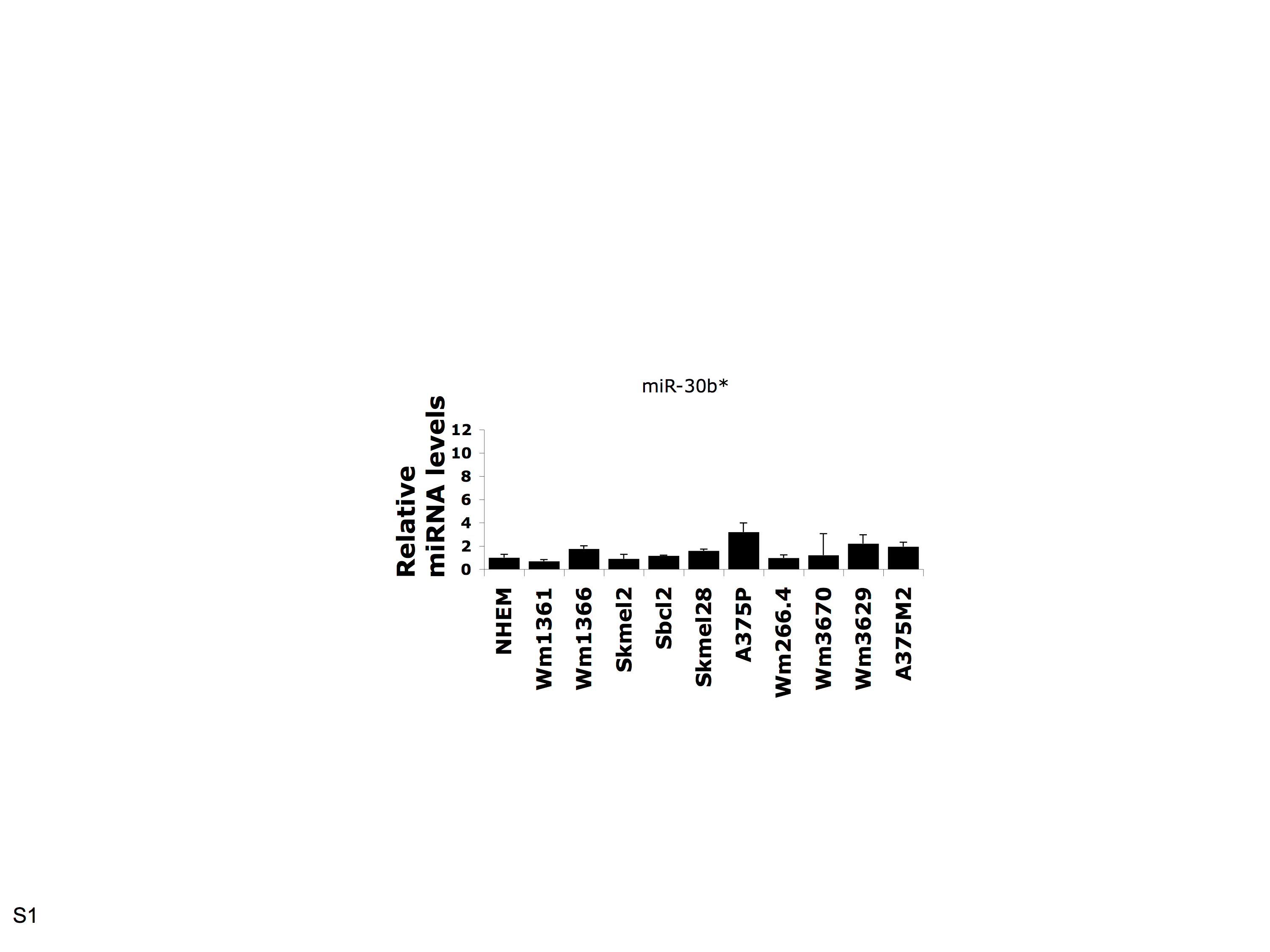

Supplement: Figure S1 — miR-30b* serves as a control for microRNA expression. Expression levels of miR-30b*, standardized to total RNA measured by Nanodrop spectrophotometry, in melanoma cell lines and normal human melanocytes. (0.39 MB TIF) [file pone.0013176.s001.tif]

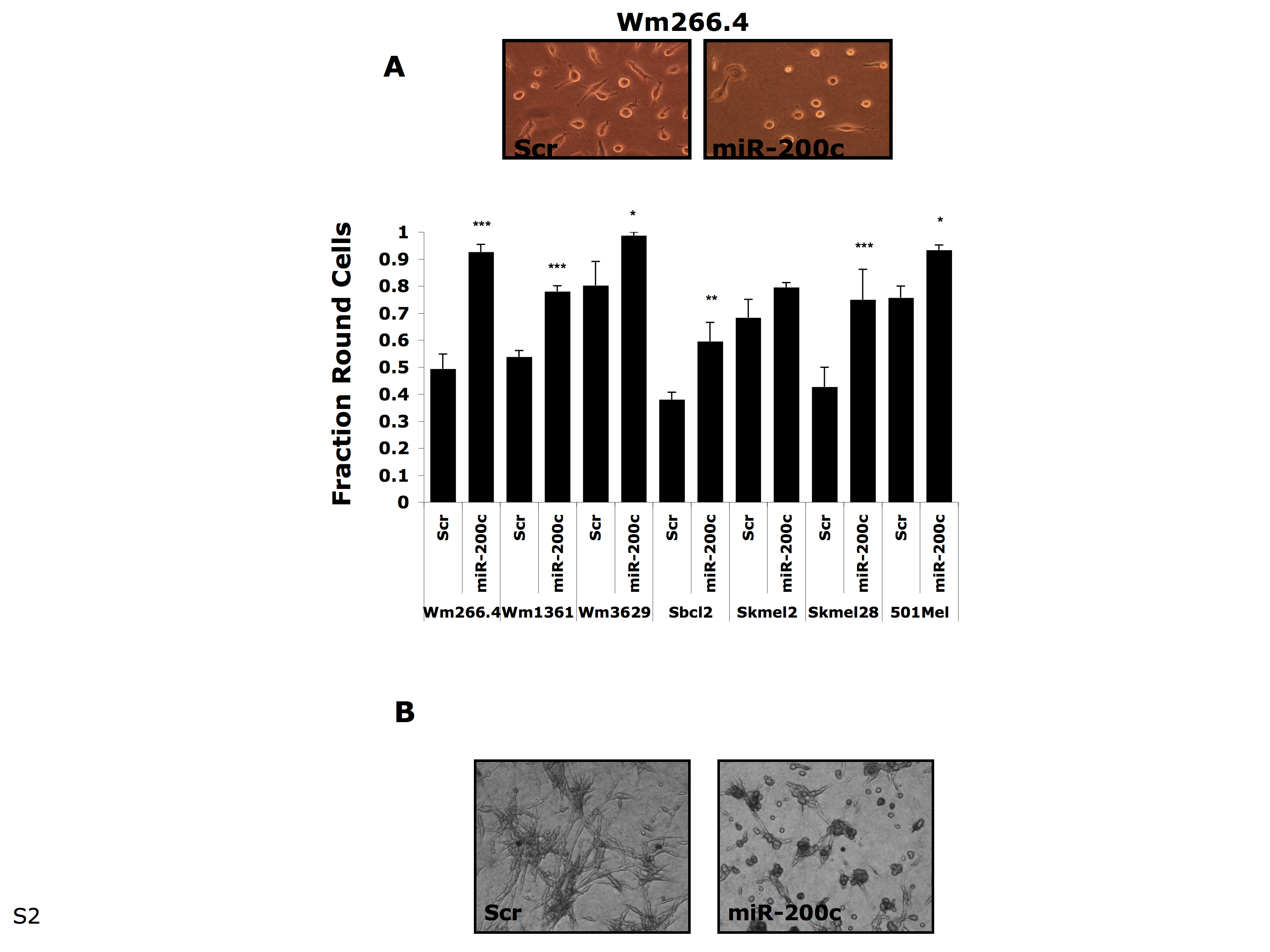

Supplement: Figure S2 — Rounded cell morphology is induced by miR-200c in melanoma cell lines and normal human melanocytes plated on a thick collagen matrix. Cells was transfected with either a negative control or miR-200c, plated on a thick collagen matrix, and examined for morphology. (A) Left Panel: Brightfield images of Wm266.4 cells. Right Panel: Quantification of the fraction of round cells following transfection with miR-200c in 7 melanoma cell lines. Error bars represent ± SEM; unpaired t-test *p<0.05, **p<0.005, ***p<0.0001. (B) Brightfield images of normal human melanocytes. (1.86 MB TIF) [file pone.0013176.s002.tif]

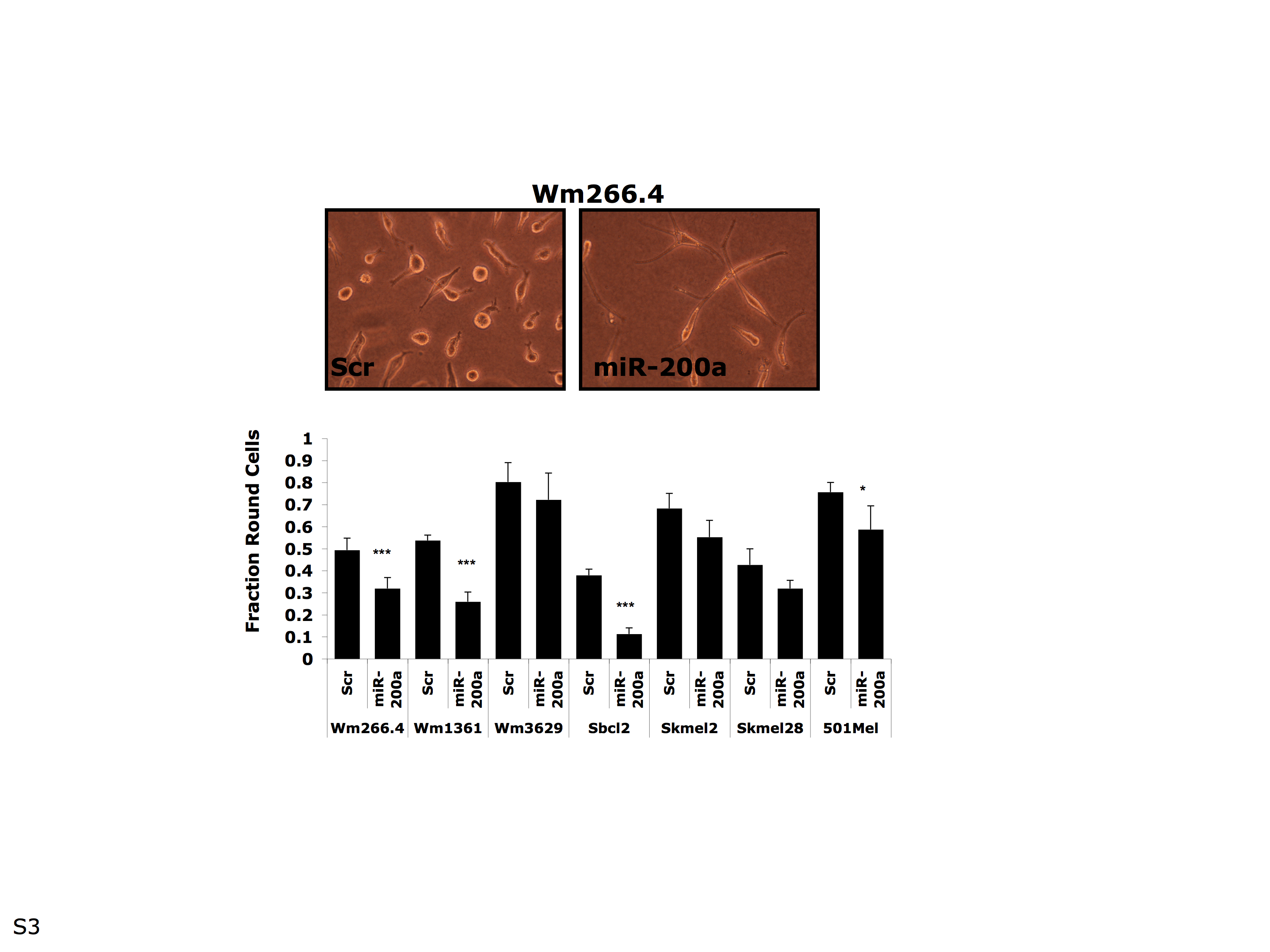

Supplement: Figure S3 — miR-200a induces elongation in melanoma cell lines on a thick collagen matrix. Cells were transfected with either a negative control or miR-200a, plated on a thick collagen matrix, and examined for morphology. Upper panel: Brightfield images of Wm266.4 cells. Lower: Quantification of the fraction of round cells following transfection with miR-200a in 7 melanoma cell lines. Error bars represent ± SEM; unpaired t-test *p<0.05, ***p<0.0005. (1.78 MB TIF) [file pone.0013176.s003.tif]

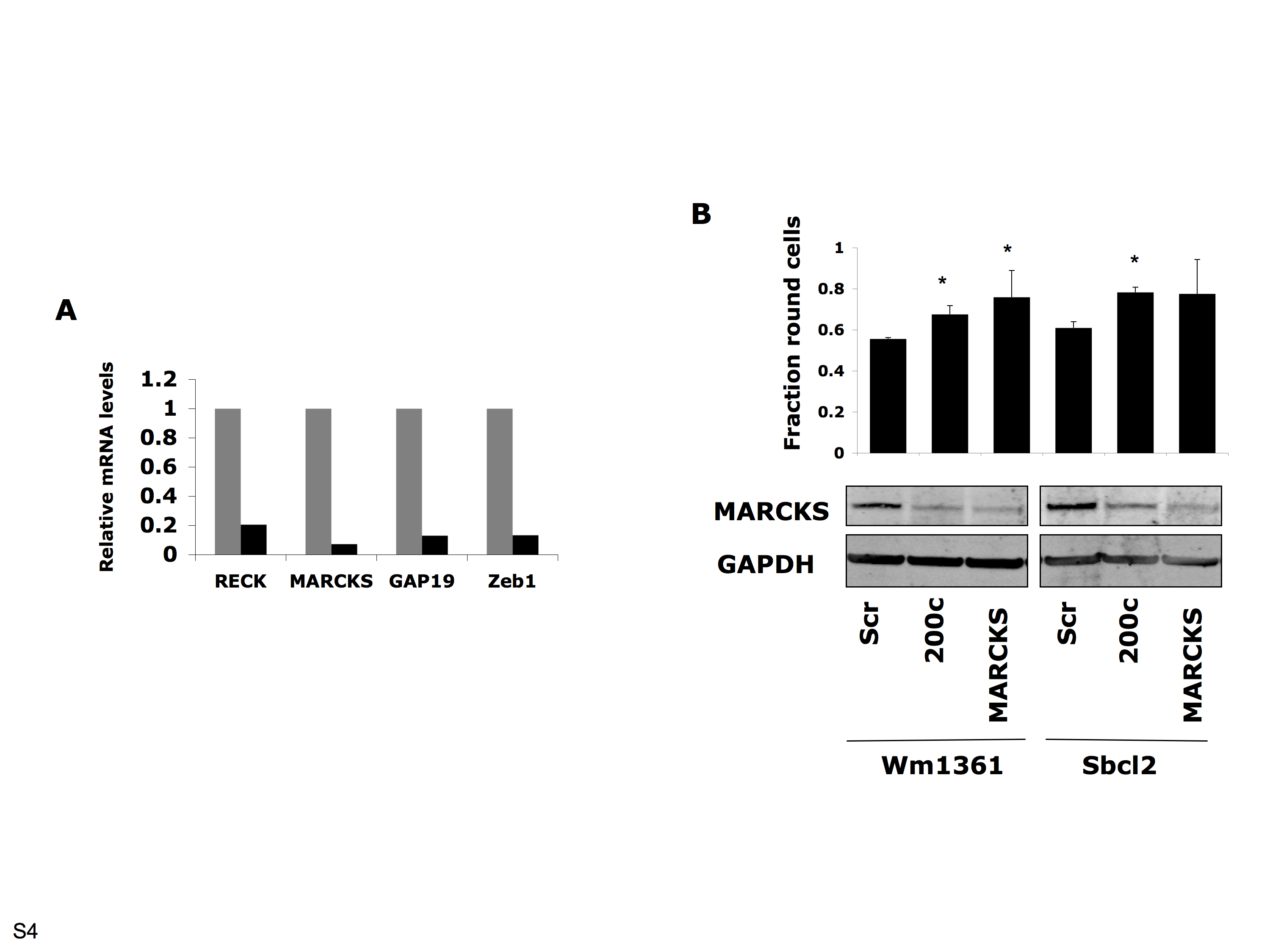

Supplement: Figure S4 — siRNA knockdown of miR-200c targets. (A) Quantitative PCR analysis, using GAPDH as a control, confirms gene knockdown by siRNAs in Wm266.4 cells imaged in Figure 2. (B) Upper: Quantification of the fraction of rounded cells following knockdown of MARCKS by siRNA or miR-200c, in Wm1361 and Sbcl2 melanoma cell lines. Lower panel: immunoblot of MARCKS expression following transfection of miR-200c or siRNA targeting MARCKS. (0.65 MB TIF) [file pone.0013176.s004.tif]
